# Supplementary figures and images for: Functional Role of Piezo1 in the Human Eosinophil Cell Line AML14.3D10: Implications for the Immune and Sensory Nervous Systems
Source: Biomolecules. 2024 Sep 14;14(9):1157. doi: 10.3390/biom14091157 (PMC11429562; doi:10.3390/biom14091157)

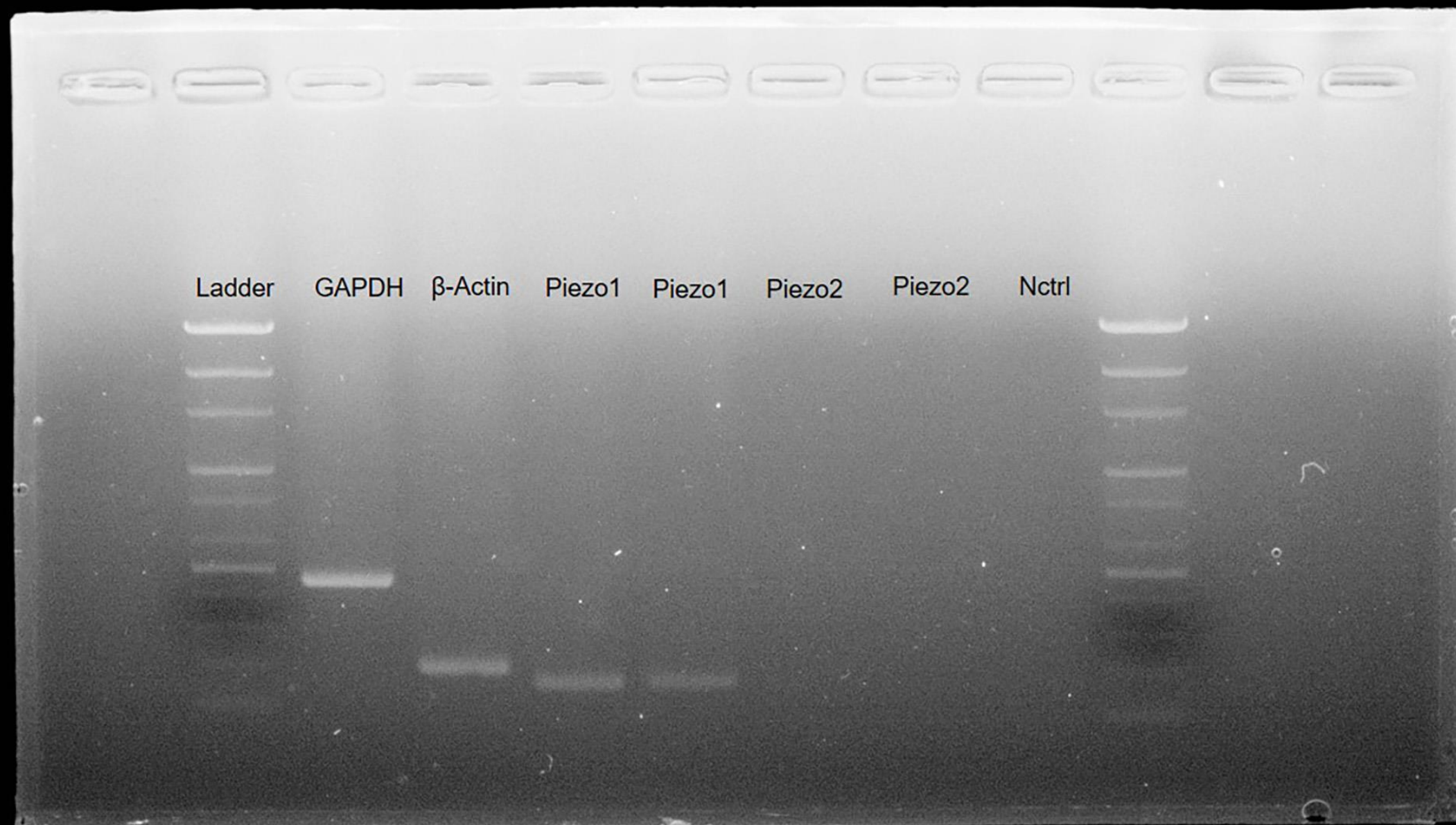

Supplement: Supplementary file 1 [file biomolecules-14-01157-s001.zip › biomolecules-3163899-supplementary.pdf]
